# Mitochondrial landscape of indigenous pig germplasm of Andaman and Nicobar Islands

**DOI:** 10.1080/23802359.2019.1660240

**Published:** 2019-09-02

**Authors:** Arun Kumar De, Perumal Ponraj, M. S. Kundu, Ramachandran Muthiyan, K. Muniswamy, A. Kundu, Dhruba Malakar, Jai Sunder, Zachariah George, Debasis Bhattacharya

**Affiliations:** a ICAR-Central Island Agricultural Research Institute, Port Blair, India;; b GCC Biotech India Pvt. Ltd., Joychandipur, India;; c National Dairy Research Institute, Karnal, India

**Keywords:** Nicobari pig, Andaman Desi pig, Mitogenome, *Sus scrofa*, Andaman and Nicobar islands

## Abstract

Nicobari pig and Andaman Desi pig are indigenous pig germplasm of Andaman and Nicobar islands, India. Over the last two decades, the pig breeds witnessed a rapid decline in population, necessitating immediate characterization and conservation. The present study depicts the complete mitochondrial genome sequence of Nicobari pig and Andaman Desi pig. The mitogenomes of both the breeds encode 37 genes including 13 protein coding genes, 22 tRNAs, and two ribosomal RNA genes. In addition, a control region (D-loop) was also present. Phylogenetic analysis showed that Nicobari is phylogenetically close to Banna mini and Breed I pig, whereas Andaman Desi pig is close to Mong cai and Jinhua pig breeds. The results of the study will be helpful for formulating of conservation strategy of the native swine breeds.

Pigs constitute a major share (27.26%) of the total livestock of Andaman and Nicobar islands and play a vital role in the livelihood of the native people of these Islands (De et al. 2014). Two genetic groups of domestic pigs namely Nicobari pig and Andaman Desi pig are available in these Islands (De et al. 2013). The population of the pig breeds has reduced drastically over the last decade and immediate conservation effort is the need of the hour. It is important to evaluate their genetic structure in order to develop the cost effective approach for their conservation (Behl et al. 2006). Moreover, the genetic root of the indigenous pig breeds is still unknown. The Mitochondrial DNA has emerged as a vital tool to study breed characterization, and phylogeography of animals (Ludwig et al. 2013; Rissler 2016). In the present work, whole mitochondrial DNA of Nicobari pig and Andaman Desi pig was sequenced and characterized for the first time by NGS based methodology. The genetic root of the pig breeds was also traced.

Blood samples were collected from the two pig breeds maintained at the institute farm of ICAR-CIARI, Port Blair (11.6234°N, 92.7265°E). DNA was isolated by standard methodology. DNA samples were stored at state repository located at ICAR-CIARI, Port Blair (voucher number CIARI_NP_1224 for Nicobari pig; CIARI_ADP_1225 for Andaman Desi pig). Enrichment of mitochondrial DNA was done. Further, library preparation was done by using Nextera XT DNA Library Preparation Kit, Illumina. The library was sequenced on a Illimina Nextseq platform with paired-end sequencing. After sequencing, the filtered reads were assembled using a reference complete mitochondrion sequence*, Sus scrofa* (Genbank accession no. NC_000845).

The complete mitochondrial DNA sequences of Nicobari pig and Andaman Desi pig were submitted to GenBank with the accession numbers MK248681 and MK248682 respectively. The mitogenome of both the pigs was 16,613 bp in length and encoded 37 genes including 13 protein coding genes (PCGs), 22 tRNAs, and two rRNAs. In addition, one A + T rich region (D-loop) was present. The order and orientation of the genes was similar to the mitogenomes of other vertebrates (Sarvani et al. 2018). All the protein coding genes except ND6 were present on heavy strand. Start codons for all the 13 PCGs were ATN codon and abbreviated/truncated stop codon was observed in ND1, ND2, COX3, ND3, and ND4.

Whole mitochondrial sequences of domestic pig breeds and wild boars were retrieved from Genbank. Phylogenetic analysis was carried out based on concatenated sequence of protein coding genes by maximum-likelihood (ML) method with 1000 bootstrap replications using MEGA-X (Kumar et al. 2018). From the phylogenetic tree (Figure 1), it was found that Nicobari pig has close phylogenetic relationship with Banna mini and Breed I pig, whereas Andaman Desi pig is close to Mong cai and Jinhua pig.

**Figure 1.**
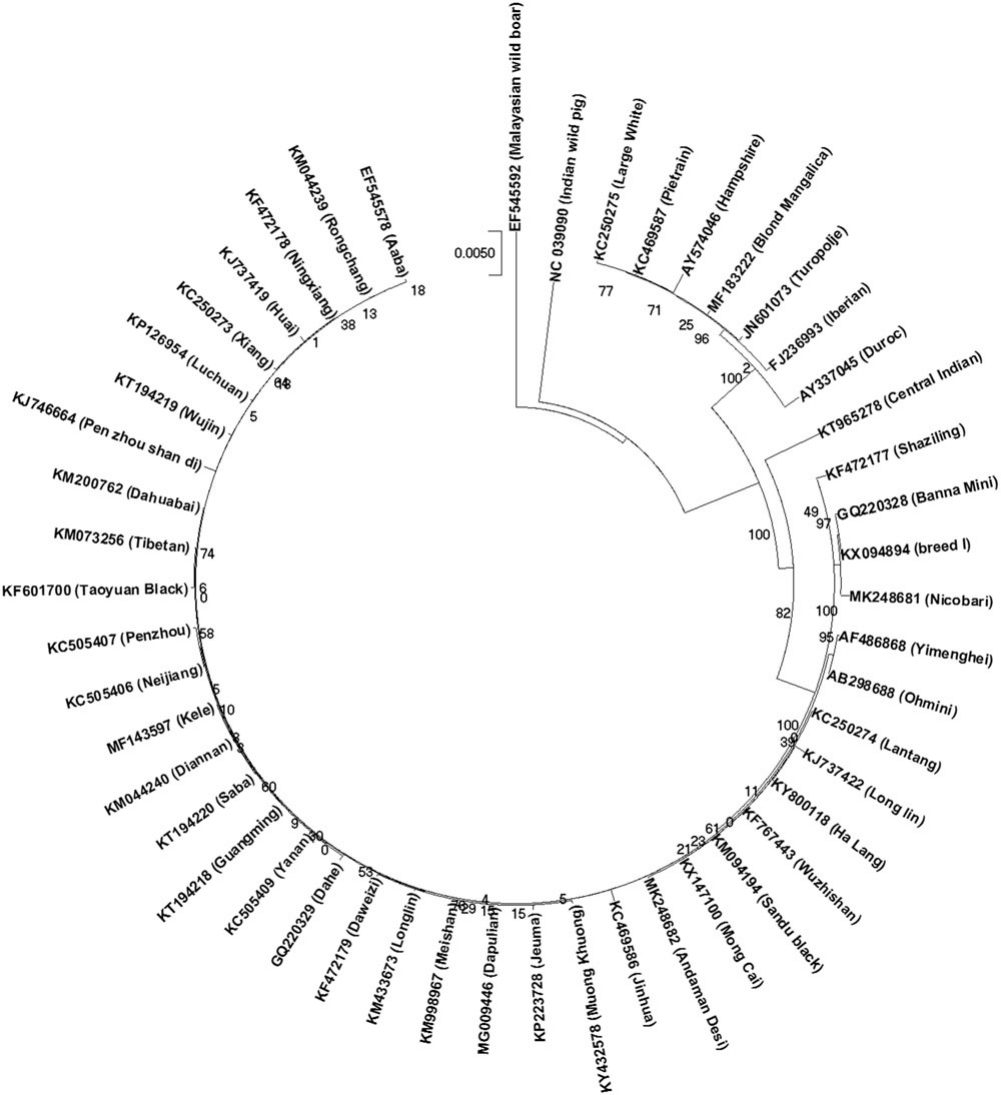
Phylogenetic analysis of indigenous pig breeds of Andaman and Nicobar islands based on concatenated sequence of PCGs. Phylogenetic relationship between mtDNA sequences of Nicobari pig, Andaman Desi pig, and other mitogenomes of porcine were analyzed using maximum-likelihood method based on the Hasegawa–Kishino–Yano model (Hasegawa et al. 1985). Accession numbers of pig breeds used are as follows: MG009446 (Dapulian), GQ220329 (Dahe), KC469586 (Jinhua), KJ746664 (Pen zhou shan di), KC469587 (Pietrain), GQ220328 (Banna Mini), KC250274 (Lantang), KC250275 (Large White), EF545592 (Malayasian wild boar), AY574046 (Hampshire), KF601700 (Taoyuan Black), KY800118 (Ha Lang), KY432578 (Muong Khuong), KX094894 (breed I), AF486868 (Yimenghei), MF143597 (Kele), KX147100 (Mong Cai), KM998967 (Meishan), KP223728 (Jeuma), KM044240 (Diannan), KM433673 (Longlin), KM094194 (Sandu black), KM044239 (Rongchang), KJ737422 (Long lin), KJ737419 (Huai), KT194220 (Saba), KT194219 (Wujin), KT194218 (Guangming), AY337045 (Duroc), KM073256 (Tibetan), KP126954 (Luchuan), KM200762 (Dahuabai), KF767443 (Wuzhishan), KC505406 (Neijiang), KC505407 (Penzhou), KC505409 (Yanan), KF472178 (Ningxiang), KF472177 (Shaziling), KF472179 (Daweizi), JN601073 (Turopolje), KC250273 (Xiang), MF183222 (Blond Mangalica), FJ236993 (Iberian), NC_039090 (Indian wild pig), EF545578 (Aaba), AB298688 (Ohmini), KT965278 (Central Indian), MK248682 (Andaman Desi), and MK248681 (Nicobari).

The current study documents the mitogenome analysis of native pig breeds of Andaman and Nicobar and results of the study will be helpful to chalk out conservation strategy of the swine breeds.1660240
